# The case against efficiency: friction in social media

**DOI:** 10.1038/s44260-025-00061-z

**Published:** 2026-01-22

**Authors:** Joshua Garland, Joe Bak-Coleman, Susan Benesch, Simon DeDeo, Renee DiResta, Jan Eissfeldt, Seungwoong Ha, John Irons, Chris Kempes, Juniper Lovato, Kristy Roschke, Paul E. Smaldino, Anna B. Stephenson, Thalia Wheatley, Valentina Semenova

**Affiliations:** 1 Arizona State University, Tempe, AZ USA; 2 University of Washington, Seattle, WA USA; 3 Santa Fe Institute, Santa Fe, NM USA; 4 Harvard University, Cambridge, MA USA; 5 Carnegie Mellon University, Pittsburgh, PA USA; 6 Georgetown University, Washington, D.C. USA; 7 Siegel Family Endowment, New York, NY USA; 8 University of Vermont, Burlington, VT USA; 9 Vanderbilt University, Nashville, TN USA; 10 University of California-Merced, Merced, CA USA; 11 Princeton University, Princeton, NJ USA; 12 Dartmouth College, Hanover, NH USA; 13 Institute for New Economic Thinking, New York, NY USA

**Keywords:** Policy, Communication, Interdisciplinary studies, Sociology

## Abstract

Social media platforms frequently prioritize efficiency to maximize ad revenue and user engagement, often sacrificing deliberation, trust, and reflective, purposeful cognitive engagement in the process. This manuscript examines the potential of *friction*—design choices that intentionally slow user interactions—as an alternate approach. We present a case against efficiency as the dominant paradigm on social media and advocate for a complex systems approach to understanding and analyzing friction. Drawing from interdisciplinary literature, real-world examples, and industry experiments, we highlight the potential for friction to mitigate issues like polarization, disinformation, and toxic content without resorting to censorship. We propose a state space representation of friction to establish a multidimensional framework and language for analyzing the diverse forms and functions through which friction can be implemented. Additionally, we propose several experimental designs to examine the impact of friction on system dynamics, user behavior, and information ecosystems, each designed with complex systems solutions and perspectives in mind. Our case against efficiency underscores the critical role of friction in shaping digital spaces, challenging the relentless pursuit of efficiency and exploring the potential of thoughtful slowing.

## Introduction

In 2004, a study by the U.S. National Institutes of Health found that “speed humps were associated with a 53% to 60% reduction in the odds of injury or death among children struck by an automobile”^1^. Introducing a small amount of friction to drivers forced them to reduce their speed and drive more attentively, which has saved lives. Speed humps are just one example of how slowing people down can lead to positive outcomes for both individuals and society. As discussed later in this manuscript, areas such as finance, neuroscience, and media consumption have all demonstrated real-world benefits from adopting a more deliberate pace before taking action. In this article, we ask what lessons society can carry over from these diverse real-world scenarios into the digital world. Are there instances where “digital speed humps,” which might slow down the user experience or the transmission of information to a broader audience, can help promote a better information ecosystem?

For most large social media companies, user engagement fuels advertising revenue—the core engine that sustains and drives their business. To capture limited attention, platforms design interfaces that keep users engaged and make content creation and consumption fast and effortless—accelerating delivery while minimizing the cognitive effort required. This often results in superficial, bite-sized content, e.g., clickbait headlines, taking precedence over more substantive content including in-depth journalism^2^.

As we discuss throughout this manuscript, both research and practical experience suggest that slowing down information consumption can lead to beneficial outcomes for individuals, platforms, and society as a whole. For example, the social media platform Nextdoor, which offers geographically-bounded online communities for real-world neighbors, has conducted multiple internal, unpublished interventions using friction to try to improve civility and decrease racist and xenophobic behavior in the posting of neighborhood warnings^3^. In one such effort from 2016, which sought to understand and mitigate racial bias in “suspicious persons” posts on the platform’s Crime and Safety category, Nextdoor changed a freeform text block into a series of questions that required users to select from among the types of activities they wished to report, with a pop-up telling users that suspicious activity reports should focus on behaviors, not appearance. The platform reinforced this with changes to the post creation flow. For example, when the user entered details about an ostensibly suspicious party’s appearance, a form requested specific details about hair, build, clothing, and shoes, and the user could not proceed if they only articulated a race. Nextdoor reported that “adding friction to the posting process helps slow people down and consider their own bias,” and found that racial profiling had decreased by 75 percent. This did, however, also come with a 50 percent increase in “abandonment” in report filing—users, in other words, at times chose not to finish filling out the form(These studies were not published but were covered extensively in the media (e.g., https://www.wired.com/2016/08/nextdoor-breaks-sacred-design-rule-end-racial-profiling/) and were verified via personal correspondence with former employees who had knowledge of the experiments.). The platform has since tried other forms of racism deterrents and “kindness” notifications to alert users to posts that, while permissible under platform policy, may be uncivil and can exacerbate tension between individuals or within communities.

The academic literature also shows similar outcomes. For example, a recent review of the literature on collective problem solving suggests that, across a wide variety of problem domains, mechanisms that prolong the diversity of solutions lead to better collective outcomes^4^. Here, frictions can slow down a group’s convergence to consensus and maintain diversity of options. For example, reductions in social interactions^5–7^ or incentivizing a reluctance to change behavior without substantial evidence^8–10^ each reduce the flow of information in ways that lead to better group-level outcomes in the long run. In addition to group-level benefits, friction also enables individuals to be more careful and deliberative—to use more “system 2” thinking as framed by Kahneman^11^. Deliberation is the kind of fundamentally human practice that techno-social engineering at times deprioritizes in favor of eliciting quick, automatic, and uniform reactions as Frischmann and Selinger explained^12,13^.

This promising approach is part of a larger set of intentional design elements known as friction-in-design (or simply friction)^13^. Friction, as we define it, refers to design decisions which encourage or oblige more effortful actions online or reduce their impact (e.g., reduced, slowed reach). Such frictions may be designed with intent to address a specific issue, as in the Nextdoor case above, or arise as an inadvertent consequence of other design constraints and affordances. We note that friction may either be made obvious to users, such as via an interstitial, or obscured so that users are unaware, such as via slowing or narrowing distribution of content on platforms. The presence of friction, to varying degrees, is an implicit aspect of design, yet little is known about the effectiveness of friction at different scales. As a result, fundamental research is needed to guide meaningful and impactful policy can be developed. Here, we review our existing understanding of frictive design and chart a course for future research.

Friction, or the lack thereof, is an implicit feature of information environments, manifesting across scales from individuals to longer-term platform dynamics. Design choices and interventions which alter any platform feature can have difficult-to-discern impacts on frictive forces as each of the scales, optimizations, connections, coupled effects, and intertwined relationships can alter outcomes. Yet this is a shared challenge of complex systems more generally, from evolved systems such as brains and animal societies through to anthropogenic systems like power grids and economies. Drawing on insights from these domains, we provide a broad complex systems perspective on friction as it relates to social media, and in particular, information consumption online. We believe this is the right approach as it will guide us and give us tools in understanding what to measure, what the holistic experimental designs should look like, and what are the possible systemic outcomes of such an intervention.

We begin with a historical perspective of some friction-based interventions by tech companies that were reported in blogs and tech reports, but received little attention in academia. We will also review much of the relevant academic literature. We link these findings with broader conceptualizations of friction across diverse discipline tasked with understanding or managing complex systems. These interdisciplinary perspectives reveal a theoretical framework for categorizing frictions which we embody in a formal state-space. Our representation aims to provide a new language and structure for describing the many different forms and functions of friction as they relate to the social media ecosystem. We then propose research designs and experiments that tie together our discussions, analysis, and state space to further our understanding of friction from an interdisciplinary perspective. We conclude with a discussion of the caveats and challenges involved in optimizing, implementing, and measuring the impact of friction.

## Background

Friction, as defined above, is a broad and at times amorphous concept, leaving room for various ways it can be implemented and interpreted. Yet simpler theoretical examples can provide an intuitive grounding for thinking about friction in unwieldy digital spaces, even if they do not necessarily align with the definition or scope we outline above. For example, Stark et al.^14^ demonstrate that in models of opinion dynamics, slowing down opinion change—by making some agents more resistant to updating—can help prevent premature polarization. Likewise, in the context of the classic voter model, Stark et al. also showed that introducing dynamic inertia, where agents are more resistant to changing opinion over time, led to a faster global consensus^15^.

Closer to policymaking, Gershenson and Helbing^16^ argue that in traffic systems, over-optimization can produce congestion and instability, whereas deliberately slower behavior enhances overall system performance. They suggest that designing for delays can increase resilience in complex systems. Similarly, in ref. ^17^ the authors examine “faster is slower” in the context of crowd dynamics and how that in the case of evacuations increased urgency can create bottlenecks and jamming, ultimately delaying exit times. These examples highlight how frictions can be implemented, which nominally increase effort for individuals but ultimately lead to efficiency gains at scale. The remainder of this section reviews literature on friction in digital environments, with particular attention to proposed typologies and experimental studies from both academia and industry.

### Typologies of friction

Friction is often examined in academic literature through the lens of typologies. Later in this manuscript, we introduce a state-space representation of friction to better analyze different types of interventions. In this section, we review existing typologies to provide context and identify ways they might complement or inform the framework we present.

A typology of “e-frictions” was developed by Tomalin to classify various types of online frictions, including those relevant to e-commerce and advertising^18^. Tomalin’s framework is structured as a hierarchical tree with nested binary classifications, such as “non-elective vs. elective” and “overt vs. covert”. While these classifications provide a valuable foundation for characterizing design frictions, they have certain limitations.

One such limitation is that frictions often exist along a spectrum rather than fitting neatly into binary categories, making it difficult to capture the nuances of their intensity. Moreover, while descriptors like “reflexive vs. transitive” are helpful for understanding the effects of frictions, our primary aim is to first characterize the frictions themselves, without deeply exploring their purpose, broader effects, or means of implementation. Moreover, the typology does not fully address aspects specific to frictions in social media design, such as whether the friction is content-specific. Addressing these gaps is essential for developing a more comprehensive understanding of frictions in this domain. Frischmann and Benesch have proposed a descriptive framework consisting of six parameters and related questions for evaluating and comparing friction-in-design measures and their effects. The parameters include the direct effect of friction on subjects, its architectural design, and its purpose. [22] Other approaches to defining friction have created taxonomies and categorizations focusing on the nature of the frictive design. For example, one recent literature review by Benedetti and Mauri on friction in the design of digital technologies presents a more comprehensive list of terms and broad classifications that highlight key aspects of frictions that researchers are exploring^19^. Several design approaches emerge from the literature, including “Slow Design,” aimed at decelerating the spread of content, and “Reflective Design,” which seeks to bring unconscious experiences into conscious awareness.

The authors also introduce a categorization of frictions into “diegetic” (on-platform) and “extra-diegetic” (off-platform). For example, a diegetic friction is illustrated by Twitter’s experiment prompting users to open articles before retweeting them, thereby discouraging uninformed sharing. In contrast, an example of extra-diegetic friction might be an interface which enables users to enhance or restrict permissions for sharing data across different platforms. These distinctions provide valuable insights into the varying contexts and mechanisms through which frictions operate. Other reviews have taken a policy perspective, offering ideas for the regulation of friction and providing policy-minded systems for classifying and comparing forms of friction, including the direct effects of friction on subjects, the purposes of friction, its scope of application, and how it is governed^13^.

In other cases, friction has been considered, defined and categorized within more specific context such as the spread of misinformation online^20^. For example, Johansson and colleagues’ review of interventions to prevent the spread of misinformation offers another approach to classifying frictions^20^. They categorize interventions based on their primary action: “contextualize,” “slow,” and “remove.” Although these terms and categorizations provide a foundational vocabulary and framework for discussing frictions, they fall short of capturing the nuances necessary to differentiate between various types of design frictions in social media. Moreover, these categorizations and schemas overlap in many ways such that the same design intervention can be interpreted through a wide variety of perspectives. For example, prompts presented to users to draw attention to accuracy would simultaneously be considered “slow”, “contextualize”, “diagetic”, “non-elective”, “overt”, and “reflexive.” Across such discourse, it may be difficult to keep track of a given design feature’s proximal (user) and emergent (system-level) effects, as well as its implementation and intended consequence. As such, a more detailed approach is needed to understand the similarities and differences between these frictions and how they operate within the specific context of social media platforms.

### Examples of industry experiments

Social media platforms have long experimented with friction through internal research. Historically, industry-produced research has been shared informally through blogs, news articles, and personal correspondence rather than academic channels. Yet in recent years, platforms such as Twitter, Facebook, Reddit, YouTube, and Nextdoor have increasingly engaged in the academic publication process, at times in collaboration with academic researchers. Many, but not all, of the reported frictions examined by industry have been visible to the user, such as interstitials or pop-ups that alert a user to a particular behavior or action. Twitter/X and Nextdoor, have run experiments with detecting when users are about to post apparently unkind or anti-social replies, and suggesting they reconsider their language^21^. Other pop-ups have sought to discourage resharing an article before opening the article, aiming to tamp down on knee-jerk reactions to headlines alone(See https://www.theverge.com/2020/9/25/21455635/twitter-read-before-you-tweet-article-prompt-rolling-out-globally-soon). In each of these cases, the platform takes no prohibitive action and instead relies on changes in user behavior to probabilistically filter content entering the network. Related but somewhat more active (from the platform’s perspective) forms of friction involve partially occluding content that may be false, or that is potentially disturbing in some way (an image of violence, for example). Here, too, users are free to view and engage with the content but are required to click to remove the occlusion and to view the content.

There are, however, visible frictions that a user cannot bypass: the dating site Coffee Meets Bagel, for example, prevents a user from making more than a certain number of connection requests per day, alerting them when they have reached their limit. Frictions which cannot be bypassed may also arise from design constraints; character limits, image size limits, and group size maximums may require revision, resizing, and creating additional groups respectively. Each of these (and other) visible, impassible features of design can have the net effect of increasing a user’s effort and providing opportunities for reflecting on whether the content should be shared.

Other forms of friction may be less obvious to the user. They may, for instance, simply be changes to the design which introduce friction but are not explicitly described as such to the user. Facebook, for example, once enabled users to invite as many other users into platform groups as they wished, simultaneously, via one invitation form. Upon observing that this was used to drive users into conspiratorial hot-button groups that the platform had decided were undesirable, the feature was disabled. Users may still invite friends to groups, but they have to go through the additional effort of doing so more granularly and over a longer time horizon(For a summary of platform related friction efforts, see https://www.wired.com/story/how-to-stop-misinformation-before-it-gets-shared/).

Similarly, it was once frictionless to share a news article or post into many Facebook groups at once, as well as to issue bulk invitations to join groups; after these affordances were abused with spam-like behavior, the sharing process came to require individual posts and invitations. WhatsApp also experimented with limiting mass message forwarding to slow down virality, particularly in regions like India where misinformation had contributed to political unrest and incidents of violence, including lynchings(For more explanation, see https://www.theguardian.com/technology/2020/apr/07/whatsapp-to-impose-new-limit-on-forwarding-to-fight-fake-news)^22^. Sometimes friction is placed on the distribution of content itself, such as slowing the dissemination of certain content online because it has been flagged as spam or untrustworthy. For example, when the U.S. Capital was attacked by a mob of Trump supporters on January 6, 2021. Facebook implemented a range of ‘break glass’ measures—some of which aimed to reduce the prevalence of political content in feeds and increase frictions around joining political groups or sharing political content.(For a summary of primary sources on this see https://www.buzzfeednews.com/article/ryanmac/full-facebook-stop-the-steal-internal-report). Frictions which manifest through algorithmic design and distribution of content will often be invisible to the user, instead exerting their effect after content has been posted. Nevertheless, they share a feature with other frictions in that the effort required for a given outcome (e.g., distributing content) is altered by design.

### Related experiments and analysis from the academy

There remains a significant opportunity to systematically investigate how different forms of friction shape human behavior in both online and offline environments through real-world or simulated experiments. These effects can be explored through a combination of agent-based simulations, field experiments, and retrospective analyses of real-world interventions. Existing work in this space has identified design strategies for implementing friction^18,23–27^, while also surfacing unintended consequences and limitations that merit careful consideration^22,28,29^, highlighting the need for integrative approaches. This growing body of work of experiments on friction currently falls into the following functionally distinct categories of frictions: cognitive frictions, which operate at the level of individual thought and deliberation; structural frictions, which alter the architecture or flow of digital systems; and normative frictions, which leverage social cues or moral framing to influence behavior. Each type of friction engages different mechanisms of influence and may be more or less effective depending on context, visibility, and user agency.

Cognitive frictions function by acting on an individual’s thought processes, disrupting automatic reactions, which is presumed to encourage more deliberate evaluation and action. One notable example of cognitive friction is the use of accuracy prompts, which are subtle prompts that ask users to reflect on the truthfulness of information before sharing it more broadly. Pennycook et al.^24^ tested this intervention by presenting participants with true and false news headlines in the style of social media posts. Participants who received an accuracy prompt were significantly less likely to indicate they would share false headlines compared to those in the control group.

In a related study, Lutzke et al.^30^ introduced friction through brief critical thinking prompts designed to activate users’ analytical reasoning when engaging with climate-related stimuli originally found on Facebook. Participants who read evaluation guidelines, or even just reflected on the importance of such guidelines, were less likely to engage with misinformation, suggesting that simple cognitive priming can redirect user attention in productive ways. Cognitive friction can also be user-inflicted, meaning users opt into interventions that constrain their own behavior in pursuit of longer-term goals. Tomalin^18^ explores this form of elective friction as an ethical strategy for content moderation. Examples include screen-time blockers, site access limits, or tools to filter inflammatory content. These forms of self-imposed cognitive friction suggest that under certain conditions, users may welcome friction as a form of digital self-regulation.

Although many experiments have found significant, promising effects of cognitive frictions, it can be difficult to map the effect sizes observed at individual scales within an experiment to the broader impacts on content at scale. One way to bridge the gap, at least theoretically, is through the use of theoretical tools. For example, Jahn et al.^29^ explored the role of cognitive friction in an agent-based simulation of misinformation spread. They found that while simple friction, such as introducing pauses or comprehension quizzes, reduced overall sharing volume, it had little impact on content quality unless paired with normative cues, such as community standards. Empirical work has echoed these findings, highlighting how even quite promising cognitive frictions may have limited overall impact on the spread of their targeted content^31,32^. For example, even though Community Notes produced large individual reductions in sharing behavior, it was nonetheless too little and too late to meaningfully alter the reach of targeted content^32^.

An alternative to cognitive frictions involves systemic or structural frictions which modify the flow of information or constrain behaviors across the entire network or platform. These structural interventions often function without users knowing it, invisibly shaping behavioral patterns indirectly by imposing limits on things such as speed, scale, or reach. One classic example comes from WhatsApp’s restriction on message forwarding. Melo et al.^22^ analyzed public WhatsApp groups and used an epidemiological model to simulate the spread of viral content. They found that sufficient reduction in the size of groups and imposing message forwarding limits can slow the rate at which misinformation spreads across the network, ultimately reducing reach.

Similarly, Jackson et al.^25^ examined how the structural design of online social networks affects the fidelity of transmitted information. Using an agent-based model, they tested the effects of placing limits on network depth (how many times a message can be reshared) and network breadth (how many people can receive it at once). Both constraints improved the ratio of true to false messages, especially under the assumption that messages degrade, via mutation or distortion, each time they are reshared. By reducing message reach and cascade length, structural friction increased the likelihood that users would encounter information closer to its original form. Together, these studies demonstrate that network-level frictions can enhance information quality while being agnostic to the content itself.

Another example of structural friction that was implemented by Facebook was to downrank content from users and groups who repeatedly posted misinformation. Vincent et al.^33^, examined the impact of this Facebook policy using data from CrowdTangle, BuzzSumo, and fact-checkers as auxiliary observation functions. They found that engagement per post in such groups drops by 16–31%, but did not observe a substantial change in posting behavior with respect to low-quality links shared within posts. Posts linking to websites with at least two articles flagged as false by fact-checkers experienced a 45% drop in engagement. While this structural friction is fairly heavy handed it illustrates the potential impact of modifying the information flow on a platform. The authors rightly suggest that understanding the impact of this kind of intervention will require collaborations between academics and industry.

Social and normative frictions function through reflection on social expectations, platform norms, and moral cues, often visible and designed to reinforce pro-social behavior. Rather than limiting technical affordances, they shape behavior by appealing to shared values or social accountability. A common example of a social or normative friction strategy involves prompting users to reconsider their actions in light of community norms. For example, as described earlier, Twitter conducted an experiment where it created a feature that warned users when their replies contained potentially offensive language, giving them the option to revise before posting^21^. Although users kept full control over whether to publish their original message, many chose to edit or abandon their replies after seeing the prompt. This kind of normative friction, in this experiment, promoted self-regulation by encouraging users to align their behavior with implicit platform values, without restricting expression.

As discussed earlier, Nextdoor’s interventions also relied on normative friction. Nextdoor used structured prompts and created constraints for their forms to encourage more thoughtful and less racially biased posts. Nextdoor’s intervention showed reductions in biased content while reinforcing the platform’s stated commitment to community safety and fairness. However, the effectiveness of normative friction depends not only on immediate behavioral outcomes but also on how the intervention is perceived by users and the long-term unintended consequences. For example, parental control technologies often introduce normative friction to steer children away from unsafe or age-inappropriate online behavior. Wang et al.^28^ found, when such restrictions are imposed unilaterally and without explanation, children often experience them as violations of their emerging autonomy which may harm the parent child relationship. However, frictions that were designed to include transparent feedback, clear justifications on why the content was being restricted, and gave opportunities for collaborative rule-setting were more likely to be seen as legitimate without harming the relationship. This example shows that even well-intentioned normative friction can have unintended consequences if it fails to respect the user’s perspective and autonomy or becomes overly paternalistic.

The examples above highlight how frictions targeting individuals (cognitive or normative), while promising, tend to less reliably achieve their intended results at scale. By contrast, more systemic design approaches to friction can have dramatic impacts. Ideally, such interventions can be applied in a content-agnostic manner, avoiding the ethical and practical challenges associated with more content-specific approaches like downranking. However, this is not always feasible—particularly in cases involving the most egregious content. Of course, this is not always possible, for example, with the most egregious content. Overall, these example experiments show that friction is not monolithic but manifests in diverse forms with distinct effects which vary across scales from individual to platforms. Beyond these forms of friction, further experimental work is also needed to explore alternative representations of friction and their impact on complex socio-technical systems.

## Friction across disciplines: lessons learned

Above we’ve surveyed a range of frictions studied by platforms and scientists alike, working by a wide variety of mechanisms and exhibiting effects spanning from individuals to cross-platform. Yet each of these interventions arose, in a sense, to address a specific problem or from consideration of a specific aspect of design. Largely divorced from a broader theory of friction in complex systems, it is possible that the space of design possibilities extends far beyond what has currently been evaluated. In this section, we evaluate friction broadly across disciplines, which we believe can offer valuable insights to expand and inform our understanding and implementation of friction in digital environments.

### Economics

Economists often prioritize efficiency—when resources are scarce, it is natural to view the time lost or opportunities forgone due to friction as a net negative. However, as we will demonstrate in this section, that intuition is not always accurate; in some cases, slowing down can actually be beneficial.

#### Financial Markets

Financial markets are a prime example where various frictions are imposed in order to ensure the stability of markets and to protect the financial well-being of certain participants. In the economics literature, the term ‘friction’ is most often used in the context of ‘search’ or ‘matching’ frictions which denote the time or cost of finding mutually beneficial trades or relationships. For example, in the labor market, search frictions may arise from incomplete or asymmetric information, or other factors that may inhibit instantaneous matching between employee and employer. Here, we use a more expansive version of the term friction to include other costs or barriers related to a broader set of transaction costs.

A simple example is ‘trading halts’, which are imposed to slow the effects of extreme price movement and volatility. Among others, the New York Stock Exchange (NYSE) outlines rules for ‘market-wide circuit breakers,’ based on the potential decline in price of the S&P 500 Index, which, if triggered, may result in a trading halt across all US exchanges for several minutes or until the next trading day. (For details, see https://www.nyse.com/network/article/nyse-increases-resiliancy-during-extreme-volatility) A similar mechanism can be triggered for specific securities if they experience a price fluctuation of a certain amount.(For details, see https://corporatefinanceinstitute.com/resources/career-map/sell-side/capital-markets/trading-halt/). The measures are taken in order to stabilize markets and prevent overwhelming losses in the face of market instability. The underlying hypothesis is that a temporary halt may allow market participants to digest any news that may have triggered the market volatility and to effectively come to a consensus on a new price. A proposed small tax on financial market transactions (the ‘Tobin Tax’) is seen by advocates as a way to curb speculation and help stock prices reflect their fundamental value^34–36^.

The markets also impose different rules on market participants - preventing certain participants from executing trades (such as retail trader 401K accounts), while requiring others to provide liquidity. Designated ‘market-makers’, for example, are required to offer to buy or sell securities within a certain price range on the latest transactions, in order to ensure the markets can provide sufficient liquidity to participants—in exchange, market makers may be offered rebates on their transaction costs and preferential treatment, in terms of order flow. On the other hand, commonly used US 401K retirement accounts often have limits on the number of trades and the amount of rebalancing that can be done within the account and policies precluding high amounts of leverage or investment in high-risk instruments (such as the selling of stock options)^37^. Retirement accounts are geared towards long-term investment strategies and prevented from exposure to tactics that may completely and instantaneously wipe-out individual savings. Even though many of the frictions listed above may preclude market participants from instantaneously executing seeming mutually beneficial trades (or may require excess trading on the part of market makers), such frictions are commonly acknowledged to improve long-term market stability and welfare of market participants.

#### Retail banking

Another prime example where frictions are deployed to prevent potentially harmful transactions is in retail banking. First and foremost, individual credit card accounts often have monthly spending limits (which are linked to the individual’s credit history), while debit accounts have overdraft limits^38^. Unusual activity may result in the freezing of an individual’s account and prevent any transactions until some identity verification is complete. Additionally, international anti-money laundering efforts require financial institutions to track and report unusual activity to government agencies—potentially temporarily precluding individuals from having access to their assets^39^. The retail banking example and the deployment of frictions, is distinct from that in the financial markets. In this case, frictions are deployed to prevent the harm caused by: identity theft, imperfect information about individual liquidity, and international crime, rather than to improve the stability of markets.

#### Labor markets

Labor markets, too, often incorporate frictions designed to improve the market functionality and/or to protect certain market participants (on top of the regular search costs and frictions that exist in these markets—see ref. ^40^ for an overview). Required layoff notifications, collective bargaining (including cooling-off periods during collective bargaining negotiations), worker-eligibility verification requirements, and various employment protection policies are all examples of frictions that are introduced into market systems with the goal of improving outcomes. It should be noted that these frictions can impact both individual outcomes at the employee-employer level, and also at the level of the broader market such as the overall rate of unemployment or degree of income inequality^41^.

#### Frictive balance in climate policy

A large literature within economics is dedicated to the study of ‘externalities’, which occur when the consumption, production, and investment decisions of individuals, households, and firms affect others that are not directly involved in the decision-making process. Externalities may be positive, when there is a difference between individual and social gain - society would be better off if the individual were to produce more of a good than dictated by market prices. They can also be negative—the case when society would be better off if less of a good was produced than dictated by the market. Although by definition, externalities aren’t shaped by focal decision-makers, frictive design provides opportunities to nonetheless shape their impact and nature.

For example, the usage and production of fossil fuels is often used as an example of an activity with a negative externality—the greenhouse externality of global warming into production and consumption decisions. Going forward, methods for mitigation of negative externalities have considered increasing friction through regulation such as imposing ‘Carbon Taxes’ to better approximate external costs^42^ for a comprehensive review). Yet reduction of negative externalities is only part of the story, and still other policies have sought to reduce friction for technologies that exert positive externalities. For example, the production of ‘green’ technologies such as efficient solar energy production directly supply power, but indirectly reduce carbon emissions in contrast to fossil-fuel based electricity production. From reducing regulatory burdens to subsidies, numerous governments have invested in easing the process of producing, distributing, deploying, and purchasing green energy technologies in order harness these externalities. Although familiar to many, this example highlights the interdependent and relative nature of competing frictive forces, the balance of which will ultimately determine outcomes of interest.

#### Key takeaways

The fundamental insight from the economic literature and practice is that imposing some transaction cost or friction can improve outcomes in various ways, including by reducing excess volatility, shifting power dynamics, facilitating legal and proper trades (including through identity verification), increasing predictability, and effectively capturing market externalities. Of course, there are also debates in the literature whether the added frictions actually achieve their stated goals when implemented, and whether there are unintended consequences of the frictions either at the individual or market level. However, in principle, the notion that less friction is always beneficial to the outcomes is not supported by the literature.

Some frictions are agency-reducing, such as the requirement of demonstrated financial sophistication to trade options. Some frictions are designed to be content agnostic, such as general circuit-breakers, where markets are halted to curb extreme panic selling, while others are specific to certain kinds of behaviors such as mass-layoff notices. In general, these frictions tend to be visible to participants, in part because they have a role in enhancing overall confidence in the smooth functioning of these systems; though there are also cases where the friction may be less visible to all participants, such as in the case of a carbon tax that may only be reflected in the broader price of a good or services.

We should also note that while frictions may be intentional, they may also be the unintentional consequence of some other intervention or design choice. Intentional frictions are most often designed to reduce harm or incentivize better outcomes; while unintentional frictions are often thought of as introducing inefficiency. However, this is not necessarily the case in systems that have not been fully optimized. As a general matter, frictions—whether intentional or unintentional—can lead to either more or less inefficiency and changes to desirable outcomes.

### Media literacy as cognitive friction

Technological systems have simplified many aspects of daily life, from workplace activities to shopping, connecting with friends and family, and even healthcare. Online tools and platforms have streamlined routines by reducing friction for processes such as making payments, booking travel and staying informed about current events. Though the learning curve for performing such tasks is not steep, there are potential negative implications for adopting new digital technologies without a firm command of their inner workings. Simplifying the process of shopping online, for example, can result in excess spending, while consuming news from unreliable sources on social media can lead to being misinformed and even stoking belief in conspiracy theories.

In a fast-paced digital environment, information consumers making rapid decisions often default to what Kahneman refers to as System 1 or Automatic System processing^11^, where they rely on mental shortcuts or heuristics to make quick judgments on the quality of information. This is the techno-social engineering that Frischmann and Selinger describe in detail—and lament^12^. In this state, people do not often stop to discern the veracity of content but instead are moved by content with high emotional valence or unusual subject matter^43^. Research has shown that such content is more likely to be shared and spread farther on social media, which can lead to broader scale misinformation adoption. In order to encourage the activation of more reflective decision-making processes—Kahneman’s System 2^11^—researchers have tested interventions designed to slow the user down by introducing friction back into the design, a sort of digital speed bump that requires the user to pay closer attention. Media literacy—the ability to access, analyze, evaluate, create, and act on communication in all forms^44,45^—helps people navigate this complex environment, but it can be negatively impacted if users lack the skills or are overly taxed by the experience.

Commonly tested interventions like nudges, roadblocks, etc. ^46^ are designed to slow the reader and, potentially, slow the spread of misinformation. These have been explored by cognitive scientists in experimental settings as well as in coordination with platform companies such as Google^47^. Such interventions have the potential to boost media literacy by giving users greater confidence and agency in engaging with content online.

Sundin^43^ notes that the fast pace of most digital media experiences, though useful, can also make users feel rushed and produce negative outcomes. The author argues that intentional friction can cause “a reflective, informative and safe behavior among the users” (p. 39). In that way, friction in design can help reduce user error by promoting a more thoughtful and methodical approach to sharing information online.

Platforms have options for incorporating design interventions into their systems that provide opportunities for more conscious consumption. Tactics such as accuracy nudges, which are prompts that remind people to consider the accuracy of online content, or labels indicating that a social media post has been fact-checked or that an image was generated with artificial intelligence, can slow the reading process, but research is mixed on their effectiveness and on whether people become inured to them^48^. More explicit friction designs include notifications that remind people to pause and read an article before sharing it, though not much is known about their efficacy^49^.

Kozyreva et al.^49^ explore a variety of interventions for mitigating misinformation throughout the communication process. The authors describe cognitive and social science interventions for social media users as “nonregulatory, nonmonetary policy measures [that] are implemented to empower people and steer their decision-making toward greater individual and public good” (pg 1046). Such interventions can be implemented by platforms or third-parties. Though the authors describe “friction” to refer specifically to an explicit type of conceptual intervention, giving the example of asking users to pause before sharing information, it can be argued that any of the interventions described in their conceptual toolbox^49^ introduce some friction into the systems by augmenting typical processes.

Boosting, in particular, is a category of cognitive intervention with the potential for a lasting media literacy benefit. In the context of misinformation, Rozenbeek et al. (2024) describe boosting as helping people to “recognize unwanted content (p. 7).”^50^. Boosting aims to help users establish or adopt longer term habits and competencies that will maximize positive user outcomes by changing the way information is presented online or providing cues that require users to think more intentionally about certain content or topics^46^. For example, a social media post containing clear, simple instructions for fact-checking a claim can help encourage that behavior^51^.

Sundin notes the design friction can offer reassurance to users that slowing down will help them complete a task more thoughtfully and without error^43^. Such interventions are helpful not only in the context of misinformation or other harmful speech, but also for typical daily tasks. Such prompts and reminders may help boost users’ confidence and, thus, their media literacy self-efficacy.

#### Key takeaways

Media literacy comprises a set of competencies users employ when engaging with all types of media. The fast-paced, overwhelming nature of online environments can impede users’ ability to activate their media literacy skills at times and may require users to expand or develop new skills. Friction design interventions built into or layered upon digital platforms that address the increased cognitive load and attention deficit may reduce rash decision-making, confusion, uncertainty, and difficulty discerning credible information that is common in online information environments. Tactics such as nudges, labels, and boosts play different roles in the communication process, but collectively they can help build and environment that better serves pro-social goals of sharing credible information.

There is a risk, however, that platform-level elements such as labels will contribute to users’ cognitive overload by adding content to an already saturated design. Design and context play a significant role in the effectiveness of AI and misinformation warning labels^52,53^. Researchers recommend designs that clearly and specifically address content authenticity to positively impact users’ social media behavior without increasing cognitive overload. Another challenge is that the effectiveness of media literacy initiatives has been shown to correlate with the audience’s level of education^54^, suggesting that media literacy’s impact may not be uniform across all demographic groups.

### Neuroscience

Friction extends beyond human-constructed domains like economics and media literacy; it also plays a vital role in biological processes, including those studied in neuroscience. Intriguingly, many of these biological mechanisms share striking similarities with the friction observed in online social systems, as we explore in this section.

#### The neuronal refractory period

The brain relies on a variety of mechanisms to maintain balance and prevent overload, and one key example of this is the neuronal refractory period. Neurons communicate through action potentials—electrical impulses that travel along their axons—but they cannot fire continuously. The refractory period is the interval required to recover from an action potential before generating the next one. These pauses, though brief, are essential for preventing overactivity. If neurons could fire in rapid succession without pause, their signals would overlap, causing confusion and potentially destabilizing the brain’s intricate signaling system. This refractory period does not merely limit the activity of a neuron; it improves the temporal precision of its signaling over time which ensures greater informational fidelity^55,56^.

There are two phases of the refractory period: absolute and relative. During the absolute refractory period, a neuron is entirely unresponsive to any incoming stimulus, regardless of its strength. The subsequent relative refractory period allows for the generation of a second action potential, but only if the stimulus is significantly stronger than usual. This dual-phase refractory mechanism ensures a controlled flow of information, preventing excessive neural activity and maintaining the stability of the brain’s signaling systems.

#### Inhibitory neurons

Inhibitory neurons contribute further to the regulation of neural activity by moderating excitatory signals. These neurons release neurotransmitters such as gamma-aminobutyric acid (GABA), which inhibit the activity of other neurons, effectively acting as a stabilizing force within the neural circuitry. In the absence of inhibitory regulation, excitatory signals could propagate unchecked, potentially leading to pathological conditions such as seizures. By releasing GABA, inhibitory neurons counterbalance excitatory inputs, ensuring that neural circuits function within a stable range and preventing excessive neural activation^57^.

#### Thalamic gating

The thalamus serves a critical role in sensory processing by acting as a selective gateway for sensory information. It filters sensory inputs, determining which stimuli are allowed to reach conscious awareness and which are disregarded. This gating function is essential in preventing sensory overload, as the brain is continuously exposed to a vast array of sensory data. Without this selective filtering, the brain would be overwhelmed by irrelevant or extraneous information. Thalamic gating enables the brain to prioritize relevant stimuli, facilitating focused attention and efficient sensory processing^58^.

#### The prefrontal cortex

The prefrontal cortex is associated with complex cognitive processes such as decision-making, planning, and the regulation of behavior. Neural activity within the prefrontal cortex supports the brain’s capacity to anticipate future outcomes, plan ahead, and align immediate actions with long-term goals^59^. Rather than simply responding to immediate stimuli, the prefrontal cortex facilitates the evaluation of competing options, weighing short-term impulses against long-term benefits. This ability to prioritize future goals in the planning process allows for more strategic decision-making, where present actions are shaped by an awareness of their future impact. Thus, the prefrontal cortex is integral to the organization of behavior that is flexible, adaptive, and future-focused, guiding actions in a way that accounts for both immediate and long-range considerations.

#### Key takeaways

The mechanisms within the brain, such as the neuronal refractory period, inhibitory neurons, thalamic gating, and the prefrontal cortex, function much like engineered friction points that optimize neural performance. These built-in “frictions” serve to reduce excess neural activity, prevent overlap of signals, and enhance the precision of communication across neural circuits. The refractory period limits how quickly a neuron can fire again, introducing a form of temporal spacing that ensures signals are not prematurely generated or inappropriately combined. Similarly, inhibitory neurons introduce controlled suppression of excitatory signals, preventing runaway activity that could lead to disorders such as seizures. Each of these mechanisms operates at different scales of neural architecture—ranging from individual neurons to large cortical systems—and though they may appear to slow or inhibit certain actions, they are essential for ensuring that the brain functions optimally. In essence, these regulatory mechanisms help the brain balance flexibility and control, ensuring it can respond to environmental demands without succumbing to excessive or chaotic activity.

The neurological examples, although perhaps at first glance distal from social media, provide many transferable insights. First and foremost, there is no universal form of friction that titrates activity across neural systems. Instead, evolved mechanisms act in concert across scales. This stands in stark contrast to existing research on social media frictions which often focuses on design and effects of friction at a given scale of organizational complexity—the user, the news-feed, platform design. Just as the impact of refractory periods on brain activity cannot be meaningfully separated from higher-order processes such s thalamic gating and descending input from prefrontal cortex, we should not expect that frictions such as post limits can be understood in isolation from algorithmic design and content moderation.

### Friction in animal collectives

Economic contexts and nervous systems reveal features of friction in the abstract—their multi-scale nature, the balance of frictive and frictionless features, and the inability to consider any given friction truly in isolation. Yet such abstractions can appear, at times, a far cry away from informational environments which may lack effective top-down control and leadership (as in economies) or shared purpose (as within a nervous system). Animal collectives, by contrast, share many of the features of online social networks—flows of information, differing incentives, true and false information spread, and at times hierarchical social structures^60–62^. Yet these systems have been shaped by natural selection, including by the incorporation of frictions, to produce effective collective action.

Fish, for example, are remarkably sensitive to risk from predation. In many prey species, cells located in the spinal cord known as “Mauthner” neurons can respond to perturbation absent any higher-level neuronal processing and fully initiate rapid startle reflexes^63^. In collectives however, this sensitivity can go awry leading to “false alarms” which threaten to spread misleading evidence of a predator across the group^60^. Yet strikingly, such false alarms rarely spread beyond a few individuals, in sharp contrast to true alarms which can recruit entire schools^60,64^. The mechanism behind this selection for true information appears to be a sort of friction—animals are evolved to respond to only a few nearest neighbors, such that the reach of alarm is short unless many fish detect the same stimulus^60^. Highlighting how principles of complex systems can illuminate similar phenomena in starkly different domains, this dynamic in fish works for the same reason that low clustering in human social networks can prevent the spread of information or behavior that requires reinforcement from multiple sources to be adopted^65^.

Beyond fish, individual attentions in many animal collectives appear to be often limited to a relatively small number of neighbors. Indeed, pigeons go so far as to preferentially respond to birds they see in their left eye alone^66^. There are strong theoretical arguments behind the evolved sparsity of animal collectives, the frictive forces this arrangement entails, and the emergent functionality. In one theoretical investigation, Mann demonstrated that sparse networks can help balance social and personal information; and that increasing the size or density of networks could lead to degraded decision-making^67^. Beyond sparsity, animal groups often have egalitarian information environments—even if members of the group exist within strong social hierarchies^62^. Equal influence in informational sharing, in a sense, becomes a balancing of frictions across individuals. Supporting this idea, Aswamenakul et al.^68^ recently used agent-based modeling of evolving decision-making collectives to show that while selection for quick decisions promoted the emergence of hierarchical organization, selection for deliberative and accurate decision-making promoted more egalitarian networks of influence.

#### Key takeaways

From the perspective of social media, these examples of frictions in animal collectives highlight how network topologies alone can produce information-filtering frictions. For animals, sparsity becomes a key feature that reduces the presence of false alarms while nonetheless permitting the spread of veritable information. These findings directly echo the research described above on WhatsApp, where forwarding limits and reduced group sizes effectively increase the sparsity of the network and encourage information to spread locally rather than globally. For fish and WhatsApp users alike, network structure appears able to tip the balance towards true information and away from falsehoods without any top-down determination of what constitutes either class. The apparent relationship between density and total friction in a system may be a general principle, leading to features such as cascading collapse in ecological and banking networks^69^.

## A state space representation of friction

**Fig. 1:**
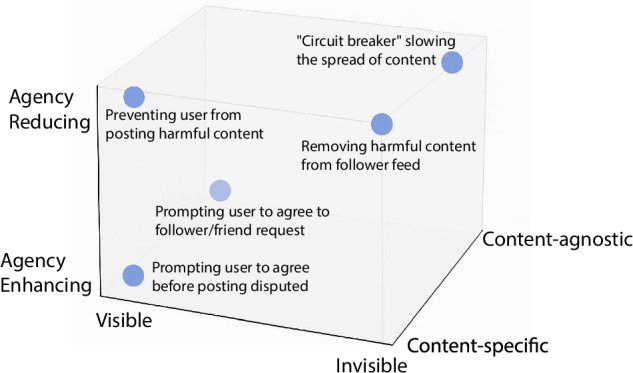
A conceptual state space representation of friction. Axes are left unquantified for two reasons: (1) friction dimensions---such as agency---can be binary (e.g., reducing vs. enhancing) or continuous (e.g., agency-guiding as a middle ground); and (2) assigning numerical values is both difficult and unlikely to improve understanding without evaluating specific metrics.

Intervening in complex social systems requires a principled framework for conceptualizing how friction is introduced through design. To that end, we propose a “state-space representation of friction” that captures the multidimensional nature of frictive interventions. This framework is not intended to be exhaustive; rather, we see it as a starting point that future studies can expand upon, potentially incorporating typologies discussed earlier. The framework is illustrated in Fig. 1, in which we outline the key attributes that describe friction in the context of social media. We argue for a state space, rather than a typology^18^, because interventions and design choices exist in a multidimensional space, exhibiting varying degrees of different friction types. We propose three key attributes that help categorize an intervention: whether it is visible or invisible to the user, whether it is content-specific or content agnostic, and whether it is agency-enhancing or agency-reducing. (For the purposes of this discussion, we consider the design choices from the standpoint of users.) Prompting a user to reconsider posting harmful content, as Twitter and Nextdoor did in their “kindness” interventions, is an example of a visible, agency-enhancing, conditional friction: the user sees the prompt and can make an informed individual choice. Temporarily slowing the spread of content which reaches too many individuals too quickly (similar to a ‘trading halt’ in the financial markets), on the other hand, would be an invisible, agency-reducing, absolute friction. Users do not directly observe why a piece of content is not reaching a wide audience, and cannot make decisions to expedite its spread.

From an experimental lens, visibility, agency-modification, and content-basis are particularly valuable dimensions because they are parameters that can be gradually adjusted in production settings. That ability makes them practically useful across a wide range of potential interventions while also aligning them fairly closely with A/B testing methods used in industry in developing complex digital experiences and enables surfacing broader ranges of insights. Therefore, the three deliver additional utility in comparison to static, descriptive useful aspects, including noting the origin of an intervention or whether it is a systematic or targeted type. In many production settings, the three are also parameters that developer teams and researchers have agency over; unlike big platform-wide changes that require complex corporate governance decision processes.

Our state space representation and outline of implementable frictions (and their individual categorization) serve two purposes. First, it offers a framework in which the merits and downside of frictions can be discussed. Second, it provides practitioners a variety of options which they can implement on their platforms, depending on their desired outcome.

Figure 1 lays out how different aspects of frictions may interact with each other, and the various implementation options that exist. Design features that incorporate frictions may or may not be visible to the users. Slowing the dissemination of certain content online, either because it is flagged as less trustworthy or because it has hit a ‘circuit breaker, (A reference to financial market “circuit breakers,” which temporarily halt trading to prevent panic selling and extreme price crashes.)’ —for example after January 6th on Facebook— may be an intervention that users do not directly observe. A dialogue box notifying users that they cannot share specific content or that they have reached the limit for the number of connections they can make in one day (as with the dating site Coffee Meets Bagel) is a visible design choice. The implementation of frictions need not reflect a top-down approach and can actually enhance user choice and freedoms (consider the popularity of apps such as Opal, Brick, or One Sec, which users install deliberately to decrease mindless and habitual screen time). Prompting users to reflect on whether something is appropriate or not before viewing, as is frequently the case with potentially Not Safe for Work (NSFW) content, enhances the user’s agency by warning them prior to accessing harmful or potentially embarrassing items. Similarly, flagging disputed content can enhance user agency by prompting them to do additional research and verify information. Frictions may or may not be agnostic to the content that is shared online. In the case of NSFW images, the friction is content-specific. However, in the case of a ‘circuit-breaker’ or a cap on the number of likes a user can have in a day, the limits are content-agnostic.

Outlining various friction characteristics can indeed help guide specific platform design choices; however, this is only part of the solution. The second, equally important component is to agree upon and standardize metrics to capture the impact that interventions with a specific friction profile might have. We suggest several categories of metrics that may be important to consider: user engagement, content quality (such as the amount of misinformation or hateful content) and overall platform experience (the ability for a platform to provide a service to users).

Establishing a state space representation of frictions to map the continuum of possible changes to social media platforms, along with metrics to compare and contrast the efficacy of different interventions, is an important step toward understanding and streamlining improvements within the social media landscape.

## Experiments

In this section, we discuss several *hypothetical* experiments aimed at examining the interaction between the introduction of friction into a system and its implications on scaling, robustness, as well as dynamics and adaptation within the framework of a complex systems lens. We believe that conducting these experiments would deepen our understanding of friction for researchers and practitioners.

### Scale of Wikipedia and Reddit communities and the diverse levels of necessary friction

While much of this paper has focused on top-down, company-driven forms of regulated friction, Wikipedia and Reddit are illustrative cases of community-based regulation, where friction and governance are enacted by users and their communities. Institutions, codes of conduct, and norms create friction in online platforms that can make it harder for misinformation and antisocial contributions to persist. On Wikipedia, hierarchies, rules, and group-based accountability systems interface with the individuals who perform edits. This structure creates frictions that disincentivize misinformation and intentional bias and encourage reliably sourced content. On Reddit, communities choose their own moderators, moderator privileges, and community rules. Understanding these self-imposed frictions and governance systems is essential to finding ways to effectively use frictions across online communities^70^.

Industry has developed a range of resources for designing community moderation systems that create friction. Notably, the Trust and Safety Professional Association offers companies and professionals a curriculum with recommendations across different aspects of friction^71^ that can help inform both practitioners and experimental researchers. In addition, the Trust and Safety Foundation hosts practitioner working groups and maintains libraries of case studies and reports that can provide useful recommendations to both industry professionals and the experimental research community^72^.

Different frictions are needed at different scales. A small community of tens of individuals will need different moderation tools, rules, and norms than a community with millions. Some work has gone into understanding how the self-regulatory aspects of communities change with community size on Reddit and Wikipedia^73,74^. Larger communities often have more complex organizational structures, involving explicit or implicit role specialization, varied posting styles, or modular subgroups. As a result, applied frictions may preferentially affect certain types of users or subgroups due variation in characteristics like visibility or network centrality. Number of moderators and rules are simple dials that can adjust the friction in these communities. Additional levers include targeting specific network clusters or posting styles.

Real-time adjustments of frictions also offer a way to study their impact. Self-regulatory frictions change over time, and companies also dynamically adjust the spread of certain content. Tracking these friction changes in time along with behavior of the individuals in the community—for example, Portuguese language Wikipedia’s CAPTCHA changes(Portuguese Wikipedia has a notable history of using technical measures—such as CAPTCHAs and IP bans—to limit contributions from non-registered users. These interventions, implemented at various points (e.g., 2008, 2013, and 2020), were accompanied by transparent community debates, all of which are unusual among large Wikipedia language editions.)—could allow us to understand how friction impacts each community. Releasing these findings and comparing across communities and platforms could enable a deeper understanding of the short and long-term effects of frictions on different types of communities.

By studying how friction is applied at scale in conjunction with the well-being of these communities, we can begin to understand what friction interventions work well and why. Could faster scaling of self-imposed frictions as communities grow lead to norms where ideas are expressed with less trolling and more adherence to reality? What kinds of top-down, structurally imposed hierarchies and regulations might be appropriate and effective at different scales? Research that examines frictions and their effects on communities is needed in order to find useful friction-based interventions across online communities.

### Resilience post intervention

Friction, like most interventions, should aim to create long-lasting effects on the system it influences. However, platforms are dynamic entities, constantly evolving through updates to interfaces, recommendation algorithms, content policies, and other features. Measuring the effectiveness of an intervention in any system involving humans is inherently complex; doing so in a non-static system that also involves human behavior presents an even greater challenge. A key question arises: how do we evaluate the impact of an intervention on human social dynamics when the underlying system is continuously changing?

Understanding what happens to the system post-intervention is critical. Does the system revert to a prior equilibrium, effectively nullifying the intended effects of the friction, or does it transition into a new, more socially beneficial steady state? These dynamics highlight the importance of designing experiments that can capture not only immediate outcomes but also the long-term impacts of intervention strategies. Such experiments are essential for assessing whether friction can serve as a viable counterbalance to the relentless drive for efficiency on online platforms, ultimately contributing to healthier and more resilient digital ecosystems.

## The case against efficiency

In this manuscript, we have presented a case against prioritizing efficiency on social media and called on the complex systems community to address this urgent challenge using an interdisciplinary approach. Social media platforms operate as interconnected “systems of systems,” characterized by complex interactions, heterogeneous community structures, varying content policies, and diverse moderation practices. These intricacies require a nuanced perspective that considers the interplay between users, platforms, and communities at multiple scales, enabling the design of interventions that promote sustainable and constructive online environments.

To support our argument, we reviewed relevant literature, examined experiments in the tech sector that demonstrate the potential impacts of friction, and highlighted the utility that many real-world systems derive from integrating friction into their design. Additionally, we outlined experiments that could investigate friction through a complex systems lens, offering pathways to better understand how friction influences platform dynamics and social behavior. Together, these discussions underscore the critical role of friction in fostering healthier and more resilient digital ecosystems by challenging the relentless pursuit of efficiency and embracing the power of deliberate slowing. To advance the study of friction, we introduced a state space representation that provides practitioners with a common framework for analyzing, comparing, and contrasting friction mechanisms across diverse domains. This representation establishes a foundation for systematically evaluating and designing friction interventions, particularly in areas where comparisons are inherently difficult yet vital.

### Caveats and challenges

While friction has many potential upsides it also presents challenges with respect to adoption and effective implementation. A central challenge in implementing friction is determining the optimal amount of friction for a desired outcome. In almost all cases, friction is not binary but extends across a spectrum of intensity. Too much friction can render a platform ineffective or unusable, while too little may lead to emergent problems that degrade user experience or trust. As many forms of friction involve human social dynamics, quantifying the impact of a particular intervention is inherently challenging—making the task of optimizing friction even more difficult. Also, different forms and examples of friction can trigger a variety of reactions. Repeated banners or messages from a platform may annoy users, for example, especially when messages are injunctive, and/or their tone is didactic. Small differences in wording can bring very different results.

In addition to these challenges, friction would not be optimized in isolation. Platforms may wish to prioritize a range of other Key Performance Indicators (KPIs) alongside friction—such as efficiency, cost, technical performance, user retention, attention, and advertising revenue. As a platform attempts to optimize or even incorporate friction these KPIs can be effected in surprising and coupled ways. For example, a platform might completely avoid friction related to content in an effort to boost user retention by preserving a perception of free speech. However, this approach could prompt advertisers to withdraw, as they may not want their brands associated with potentially harmful or controversial content. It may also affect user retention and attention as users may choose not to engage with toxic content. Identifying an optimal level of friction in a socio-technical system with competing interests is likely intractable, yet it remains a critical area for further research if progress is to be made^75^. In the absence of a straightforward measure for system-wide optimization, friction must be carefully studied through well-structured interdisciplinary experimentation between academics and industry like those outlined in this perspective.

There are other key challenges that warrant further investigation. For instance, friction-based interventions may have differing short- and long-term effects. Mehta^76^ explored this by examining the effect of slowing down both high- and low-credibility news articles, finding discrepancies between short and long term outcomes for both high- and low-credibility news. This illustrates the need for longitudinal studies to better understand the down stream effects of this kind of intervention.

It is also important to acknowledge that while friction may mitigate some of the harms associated with social media platforms, its potential to drive broader systemic change in society remains uncertain. For example, friction can slow the spread of hateful rhetoric, but it cannot eliminate racism. It may reduce the circulation of false information, but it will not erase the existence of conspiracy theories. Though extraordinarily challenging, understanding whether—and to what extent—friction can contribute to deeper societal change should be a central focus of future research.

As we have discussed throughout this perspective, frictive interventions should aim to produce lasting, meaningful outcomes. Designing durable interventions requires not only mechanisms to measure their immediate impact, but also longitudinal studies to assess whether those effects persist over time. As noted earlier in this section, drawing reliable inferences in the context of non-stationary human social dynamics is incredibly challenging. Without this capacity, designing interventions that are both impactful and durable becomes significantly more difficult. As such, developing and evaluating these measures should be a key area for future research—one that is especially well-suited for contributions from the complex systems community.

## Data Availability

No datasets were generated or analysed during the current study.
